# Patients with Heart Failure and Preserved Ejection Fraction Are at Risk of Gastrointestinal Bleeding

**DOI:** 10.3390/jcm8081240

**Published:** 2019-08-17

**Authors:** Lore Schrutka, Benjamin Seirer, Franz Duca, Christina Binder, Daniel Dalos, Andreas Kammerlander, Stefan Aschauer, Lorenz Koller, Alberto Benazzo, Asan Agibetov, Marianne Gwechenberger, Christian Hengstenberg, Julia Mascherbauer, Diana Bonderman

**Affiliations:** 1 Division of Cardiology, Medical University of Vienna, A-1090 Vienna, Austria; 2 Division of Thoracic Surgery, Medical University of Vienna, A-1090 Vienna, Austria; 3 Center for Medical Statistics, Informatics and Intelligent Systems, Medical University of Vienna, A-1090 Vienna, Austria; 4 Department of Internal Medicine II, Medical University of Vienna, Waehringer Guertel 18-20, A-1090 Vienna, Austria

**Keywords:** heart failure, oral anticoagulation, bleeding, hemodynamics

## Abstract

Aims. Two thirds of patients with heart failure and preserved ejection fraction (HFpEF) have an indication for oral anticoagulation (OAC) to prevent thromboembolic events. However, evidence regarding the safety of OAC in HFpEF is limited. Therefore, our aim was to describe bleeding events and to find predictors of bleeding in a large HFpEF cohort. Methods and Results. We recorded bleeding events in a prospective HFpEF cohort. Out of 328 patients (median age 71 years (interquartile range (IQR) 67–77)), 64.6% (*n* = 212) were treated with OAC. Of those, 65.1% (*n* = 138) received vitamin-K-antagonists (VKA) and 34.9% (*n* = 72) non-vitamin K oral anticoagulants (NOACs). During a median follow-up time of 42 (IQR 17–63) months, a total of 54 bleeding events occurred. Patients on OAC experienced more bleeding events (*n* = 49 (23.1%) versus *n* = 5 (4.3%), *p* < 0.001). Major drivers of events were gastrointestinal (GI) bleeding (*n* = 18 (36.7%)]. HAS-BLED (Hypertension, Abnormal Renal/Liver Function, Stroke, Bleeding History or Predisposition, Labile INR, Elderly, Drugs/Alcohol Concomitantly) score (hazard ratios (HR) of 2.15 (95% confidence interval (CI) 1.65–2.79, *p* < 0.001)) was the strongest independent predictor for overall bleeding. In the subgroup of GI bleeding, mean right atrial pressure (mRAP: HR of 1.13 (95% CI 1.03–1.25, *p* = 0.013)) and HAS-BLED score (HR of 1.74 (95% CI 1.15–2.64, *p* = 0.009)] remained significantly associatiated with bleeding events after adjustment. mRAP provided additional prognostic value beyond the HAS-BLED score with an improvement from 0.63 to 0.71 (95% CI 0.58–0.84, *p* for comparison 0.032), by C-statistic. This additional prognostic value was confirmed by significant improvements in net reclassification index (61.3%, *p* = 0.019) and integrated discrimination improvement (3.4%, *p* = 0.015). Conclusion. OAC-treated HFpEF patients are at high risk of GI bleeding. High mRAP as an indicator of advanced stage of disease was predictive for GI bleeding events and provided additional risk stratification information beyond that obtained by HAS-BLED score.

Clinical Perspectives: A general clinical observation is that HFpEF patients on OAC are often hospitalized due to gastrointestinal bleeding or other major bleeding events. Beyond the well-established HAS-BLED score, right-sided filling pressure—as a marker of HFpEF severity—seems to be an independent predictor of gastrointestinal bleeding.

Translational Outlook: A better understanding of the pathophysiology underlying the observed association between high right atrial pressures in HFpEF patients and gastrointestinal bleeding could lead to improvement in patient care. Larger trials are warranted to guide patient-tailored anticoagulation therapy in HFpEF patients.

## 1. Introduction

Heart failure with preserved ejection fraction (HFpEF) accounts for half of all heart failure (HF) cases and is associated with considerable morbidity and mortality rates [1,2,3,4]. Atrial fibrillation (AF) is present in approximately two thirds of HFpEF patients [5]. Both conditions are considered as age-related diseases and are, as such, emerging as a new cardiovascular epidemic with their coexistence carrying a particular adverse prognosis [5].

In order to prevent thromboembolic events in this high-risk patient population, current guidelines recommend the use of oral anticoagulants (OAC) [6]. Vitamin K antagonists (VKA) and non-vitamin K oral anticoagulants (NOACs) have been shown to reduce thromboembolic events, but long-term use puts certain patients at higher risk for serious bleeding events [7,8,9,10,11]. Therefore, precise risk stratification for both thromboembolic and bleeding risk is of high priority in order to identify patients in whom anti-thrombotic therapy would achieve maximum treatment benefit with the lowest risk of complications. However, finding such a balance is challenging, since factors known to increase the risk of stroke have also been identified to increase the risk of bleeding [12].

To provide a remedy, several clinical factors have been incorporated into risk stratification systems to better define thromboembolic and bleeding risk. Of the many available risk-stratification tools, current guidelines recommend the use of the CHA_2_DS_2_-VASc (Congestive heart failure, Hypertension, Age ≥75 years, Diabetes mellitus, prior Stroke, Vascular disease, Age 65–74 years, Sex category) score [13] to estimate the stroke risk and the HAS-BLED (Hypertension, Abnormal Renal/Liver Function, Stroke, Bleeding History or Predisposition, Labile INR, Elderly, Drugs/Alcohol Concomitantly) score [14] for bleeding risk. Beyond recommendations, these scores have proven best predictive accuracy [15].

Yet, the body of evidence regarding efficacy and safety of OAC as well as specific risk markers for bleeding in HFpEF cohorts is limited. Since bleeding risk seems to be higher in HF patients as compared to non-HF controls [16], and a history of HF is rather a predictor of major bleeding than of thromboembolic risk [17], we aimed to describe bleeding events and find predictors of future hemorrhage in a large HFpEF cohort.

## 2. Methods

### 2.1. Subjects and Study Design

Consecutive patients presenting with HFpEF between December 2010 and June 2018 were prospectively included in an observational registry established at the Department of Cardiology of the Medical University of Vienna. Written informed consent was collected from all patients before enrollment in the institutional registry. The study protocol complied with the Declaration of Helsinki and was approved by the local Ethics Committee (EK #796/2010). Data on bleeding events were collected over a median follow-up time of 42 (interquartile range (IQR) 17–63) months.

### 2.2. Clinical Definitions

HFpEF was diagnosed according to the current consensus statement of the European Society of Cardiology [18] and the American College of Cardiology Foundation/American Heart Association task force [19]. The following diagnostic criteria had to be fulfilled for study inclusion: (I) clinical signs and symptoms of HF. Symptoms of HF included requirement of treatment with diuretics or current symptomatic HF (New York Heart Association (NYHA) classes II–IV); (II) echocardiographic left ventricular (LV) ejection fraction ≥50%; (III) relevant structural heart disease (LV hypertrophy and/or left atrial enlargement) and evidence of diastolic LV dysfunction on echocardiography; and (IV) serum N-terminal pro–B-type natriuretic peptide (NT-proBNP) concentrations ≥220 pg/mL [18]. For confirmation of diagnosis, right heart catheterization (RHC) was performed in all patients. HFpEF was confirmed when pulmonary arterial wedge pressure (PAWP) exceeded 12 mmHg [18]. Baseline evaluation included physical examination, 12-lead electrocardiogram, laboratory assessment including serum NT-proBNP measurement, transthoracic echocardiography (TTE), coronary angiography, and cardiac magnetic resonance (CMR) imaging.

AF was ascertained based on at least one in-hospital and/or ambulatory diagnosis of AF. The presence of comorbidities was recorded according to the respective guidelines [20,21]. HAS-BLED bleeding risk scheme for AF [14] and CHA_2_DS_2_-VASc (Congestive heart failure, Hypertension, Age ≥75 years, Diabetes mellitus, prior Stroke or TIA or thromboembolism, Vascular disease (e.g., peripheral artery disease, myocardial infarction, aortic plaque), Age 65–74 years and Sex category (i.e., female sex)) score for stroke risk in AF [13] were calculated for each patient.

### 2.3. Outcome Measures and Follow-Up

Patients were prospectively followed by outpatient visits and/or telephone calls at 6-month intervals. The primary outcome measure of this analysis was major bleeding occurring within the follow-up period. In accordance to the definitions recommended by the International Society on Thrombosis and Haemostasis [22] major bleeding was defined as fatal bleeding and/or bleeding into a critical organ (intracranial, retroperitoneal, pericardial, or intramuscular) and/or clinically relevant bleeding with a drop in hemoglobin ≥2 g/dL or requirement of transfusion.

### 2.4. Diagnostic Modalities

Conventional TTE was performed by board-certified physicians using high-end scanners (Vivid E9 and Vivid 7; General Electric Healthcare, Chicago, IL, USA) according to standard protocols [23]. Patients without contraindications (e.g., use of an intracardiac device, advanced renal failure) also underwent CMR on a 1.5-T scanner (Avanto; Siemens Medical Solutions, Erlangen, Germany).

Hemodynamic measurements were performed at baseline using a 7 F Swan-Ganz catheter (Edwards Lifesciences GmbH, Vienna, Austria) via femoral access. LV enddiastolic pressure (LVEDP) measurements were performed using a 5 F pigtail catheter (Cordis, Milpitas, CA, USA). PAWP, systolic pulmonary artery pressure (sPAP), diastolic pulmonary artery pressure (dPAP), and mean pulmonary artery pressure (mPAP), as well as mean right atrial pressure (mRAP) were documented. Cardiac index was measured by thermodilution and by Fick’s method. The diastolic pulmonary vascular pressure gradient was calculated as the difference between dPAP and PAWP during pullback. Pulmonary vascular resistance was calculated by dividing the transpulmonary gradient by cardiac output.

### 2.5. Statistical Analysis

Continuous data were presented as median and IQR and discrete data were presented as counts and percentages. Spearman’s rank correlation coefficient was used to estimate correlations between continuous variables. Comparisons between groups were performed using Chi-square test or Fisher’s test.

Cox proportional hazard models were applied to assess the effect of the respective variables on primary outcome. Univariable Cox regression model was performed for each variable of interest. Adjusted hazard ratios (HR) were obtained by including each factor that reached statistical significance (*p* < 0.05) in univariate analysis, by a forward selection procedure. Kaplan–Meier curves were plotted using dichotomized variables above and below the median and compared using the log-rank test. Harrell’s C-statistic was used to assess the predictive value of the respective variables for the primary outcome. Category-free net reclassification improvement (NRI) and integrated discrimination improvement (IDI) were calculated to estimate an improvement in individual risk prediction. Two-sided *p*-values < 0.05 were used to indicate statistical significance. A false discovery rate of 0.05 was chosen to correct for multiple testing errors in the Cox regression analysis using the Benjamini and Hochberg procedure. SPSS 18.0 (IBM SPSS, Armonk, NY, USA) and STATA 15 (StataCorp LLC, College Station, TX, USA) were used for analyses.

## 3. Results

### 3.1. Clinical Baseline Characteristics

A total of 328 patients (median age 71 years (IQR 67–77); 71.0% female) diagnosed with HFpEF were prospectively enrolled in our registry. Detailed clinical baseline characteristics are displayed in Table 1; 212 (64.6%) of all patients were treated with OAC, with 192 (90.6%) patients having documented AF and 20 (9.4%) being treated for preceding thromboembolic events. Of those on OAC, 138 (65.1%) received a VKA and 74 (34.9%) received a NOAC.

Patients treated with OAC were significantly older (*p* = 0.026), had lower systolic blood pressures (*p* = 0.005), presented with more advanced stages of NYHA functional classes (*p* = 0.030), and on an average they had higher NT-proBNP values (*p* < 0.001) as compared to the remaining patients. Anticoagulated patients had higher CHA_2_DS_2_-VASc scores (*p* < 0.001). Median HAS-BLED score was comparable between both groups (*p* = 0.635). Additionally, they had statistically lower serum cholinesterase levels (*p* = 0.030), higher aspartate aminotransferase (*p* = 0.001), gamma-glutamyltransferase (*p* < 0.001) and creatinine levels (*p* = 0.003; Supplementary Table S1).

### 3.2. Structural Characteristics of the Heart and Hemodynamic Parameters

Echocardiographic measurements showed that patients on OAC had larger left and right atria (both *p* < 0.001), a higher proportion of moderately to severely reduced right ventricular (RV) performance (*p* = 0.001), higher sPAP (*p* < 0.001) and a higher proportion of significant tricuspid regurgitation (*p* < 0.001; Supplementary Table S1). Additionally, CMR imaging confirmed higher left and right atrial indices (both *p* < 0.001), lower RV ejection fractions (*p* = 0.022) and a trend towards higher RV end-diastolic volume indices (*p* = 0.056; Supplementary Table S1). In line with this, invasive hemodynamic measurements showed higher mPAP (*p* = 0.049) as well as higher PAWP (*p* = 0.006) and lower cardiac indices (*p* = 0.011; Table 1).

### 3.3. Bleeding Events

During a median follow-up time of 42 (IQR 17–63) months, a total of 54 bleeding events were recorded. Overall, patients on OAC experienced more bleeding events than the remainder of the cohort (*n* = 49 (23.1%) versus *n* = 5 (4.3%), *p* < 0.001) (Table 2). Major drivers of registered events were gastrointestinal (GI) bleeding (*n* = 18 (36.7%)). Other events registered were hematoma, cerebral, nasal, urogenital and other bleeding such as hemoptysis or hemorrhagic pericardial effusion (Table 2). Among those who were orally anticoagulated with VKA, a higher incidence of bleeding was recorded compared to patients receiving NOACs (*n* = 33 (23.9%) versus *n* = 16 (21.6%), *p* < 0.001).

Three bleeding events were fatal and all of them were associated with an overdose of VKA. Transfusions were necessary in 10 cases, 9 of these due to GI bleeding and one intramuscular bleeding event. 6 of these patients have been treated with VKA and 4 with NOACs. The overall incidence of thromboembolic events was 2.0% (*n* = 6).

### 3.4. Predictors of Overall Bleeding

To identify predictors of bleeding, the Cox proportional hazard model revealed the HAS-BLED score as the strongest predictor for overall bleeding with a HR of 2.23 (95% CI 1.72–2.89, *p* < 0.001) after multivariable adjustment (Supplementary Table S2).

When analyzing patients treated with OAC, univariate Cox regression revealed that parameters derived from invasive hemodynamic measurements were predictive for bleeding events beyond predictors seen in the first model (Table 3). Namely, mRAP (HR of 1.05 (95% CI 1.01–1.11, *p* = 0.031)) and PAWP (HR of 1.06 (95% CI 1.00–1.12, *p* = 0.041)) were predictive in the univariate model. However, both parameters lost their predictive power after multivariable adjustment.

### 3.5. Predictors of Gastrointestinal Bleeding

Due to the high incidence of GI bleeding observed in OAC-treated patients [*n* = 18 (8.5%)] further subgroup analysis was performed. When comparing clinical characteristics of patients experiencing GI bleeding with those who did not, we were not able to detect a difference in CHA_2_DS_2_-VASc score (5 versus 5, *p* = 0.289; Supplementary Table S3). However, patients with GI bleeding had higher HAS-BLED scores (4 versus 3, *p* < 0.001). Additionally, affected patients had higher mPAP [40 mmHg (IQR 33–46) versus 32mmHg (IQR 26–38), *p* = 0.010], higher mRAP [16 mmHg (IQR 11–19) versus 11 mmHg (IQR 8–15), *p* < 0.001] as well as higher PAWP [26 mmHg (IQR 20–29) versus 19 mmHg (IQR 16–23), *p* = 0.001] in invasive hemodynamic measurements.

Univariable regression analysis showed a significant association of the invasively measured hemodynamic parameters with GI bleeding including mPAP, mRAP, PAWP and LVEDP in OAC-treated patients (Table 4). After multivariable adjustment, only mRAP and HAS-BLED score remained independent predictors of GI bleeding events with a HR of 1.13 (95% CI 1.03–1.25, *p* = 0.013) and a HR of 1.74 (95% CI 1.15–2.64, *p* = 0.009), respectively.

**Figure 1:**
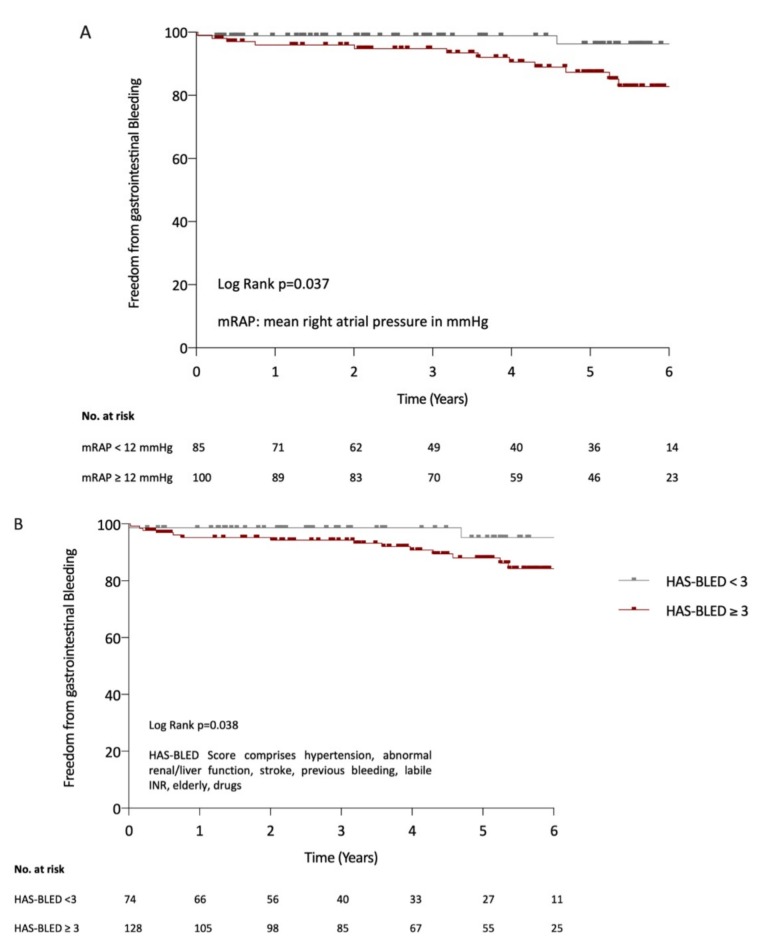
Kaplan–Meier curves for gastrointestinal bleeding events according to mean right atrial pressure (mRAP) (**A**) and the HAS-BLED score (**B**).

Kaplan–Meier analysis demonstrated significantly more GI bleeding events over time in patients with higher mRAP (Figure 1A; *p* = 0.037, log-rank test), and higher HAS-BLED scores (Figure 1B; *p* = 0.038; log-rank test). In detail, freedom from GI bleeding in patients with mRAP ≥ 12 mmHg was present in 55.2% versus 71.5%, and in 50.1% versus 95.5% of patients with HAS-BLED-scores ≥3 after 6 years of observation.

C-statistic revealed predictive ability with AUC of 0.71 (95% CI 0.60–0.83) for mRAP and 0.63 (95% CI 0.49–0.78) for HAS-BLED score. The combination of mRAP and HAS-BLED score revealed an improvement in C-statistic of 0.71 (95% CI 0.58–0.84, p for comparison 0.032). The additional prognostic value beyond that assessable with HAS-BLED score was confirmed by significant improvements in NRI (61.3%, *p* = 0.019) and IDI (3.4%, *p* = 0.015).

## 4. Discussion

In the present study we describe the incidence of bleeding events in a well-characterized cohort of HFpEF patients consecutively enrolled in our specialized outpatient clinic for HFpEF. A high proportion of orally anticoagulated patients experienced major bleeding events with a majority attributable to GI bleeding. When analyzing risk factors for GI bleeding in patients with OAC, only HAS-BLED score and invasively measured mRAP remained independent predictors of future events.

Although approximately two thirds of HFpEF patients have an indication for OAC, the body of evidence regarding efficacy and safety of OAC as well as specific risk markers for bleeding in HFpEF cohorts is limited. Bleeding risk has been identified to be higher in HF patients as compared to non-HF controls [16], and a history of HF is rather a predictor of major bleeding than of thromboembolic risk [17]. Our cohort displayed high yearly major bleeding rates of 3.2% in NOAC-treated and 4.1% in VKA-treated patients when compared to pivotal NOAC trials [7,8,9,11]. These rather high bleeding rates are in line with the fact that patients in our cohort were highly comorbid, and classical risk factors for bleeding events were more pronounced than in other patient populations. In fact, clinical hallmarks of HFpEF patients are advanced age, chronic kidney disease and liver function abnormalities, all of which are independent components of the HAS-BLED score. In line with this, HAS-BLED score proved to independently predict bleeding risk in patients analyzed in our study.

However, the high proportion of bleeding events originating from the upper or lower GI tract was striking (2.2% in NOAC and 2.1% in VKA). While the HAS-BLED score remained the strongest predictor of future bleeding events in the overall HFpEF cohort as well as in the subgroup of orally anticoagulated patients, this did not hold true for GI bleeding, where mRAP outperformed the predictive value of the HAS-BLED score.

Several pathophysiological explanations regarding the strong predictive ability of mRAP on the event rate of GI bleeding may be put forward. Backward transmission of elevated filling pressures from the LV into the pulmonary circulation and right-sided heart chambers may finally lead to supranormal blood pressures in the splanchnic venous system and thereby trigger bleeding events in pre-existing GI pathologies. Another plausible link between mRAP and bleeding from the GI tract is the presence of a chronically congested liver with consecutively impaired synthesis of clotting factors. However, it is well established in the literature [24] that impaired synthesis of clotting factors is counterbalanced by a parallel reduction in anti-thrombotic factors. In summary, the link between mRAP and GI hemorrhages cannot entirely be elucidated. There seems, however, to be an analogy between the observed findings in the low-pressure circulation and the well-known association of high blood pressure and hemorrhagic stroke in the systemic circulation [25].

Irrespective of potential mechanistic explanations, our findings are of high clinical relevance and should prompt a detailed risk assessment for GI bleeding in this highly vulnerable patient population. Bleeding risk is highly dynamic and may increase with progression of the underlying disease. Therefore, regular re-assessment of modifiable bleeding risk factors may become a pivotal safety measure. Our data indicate that HFpEF patients requiring OAC therapy should be closely monitored. Additional data are needed to guide therapeutic consequences such as dose reduction or even left atrial auricular closure in order to direct or tailor therapy in specific patient sub-populations and in specific care contexts.

## 5. Limitations

This study is not free of limitations. First of all, data on clotting factors and coagulation status prior to OAC initiation could have been of interest but were not available. Secondly, whether the underlying bleeding originates from telangiectasia in analogy to the well-described Heyde syndrome [26] or other pathologies remains unknown, since systematic information from endoscopies is missing. Third, due to the single-center nature, a center-specific bias cannot be excluded. In fact, data from a recently published administrative database [27] did not show any difference with respect to bleeding events between HFpEF patients, HF patients with reduced ejection fraction or unaffected controls. However, HF diagnosis and classification were simply based on ICD-9 codes, and the authors could not exclude that the observed findings could be attributed to a potential misclassification of patients with symptomatic AF, but without true HF, as HFpEF. Our study, by contrast, comprises a well-characterized HFpEF cohort. All patients underwent a comprehensive clinical work-up with invasively measured confirmation of elevated LV filling pressures. Furthermore, patients in our study were in relatively advanced stages of HF with elevated pulmonary pressures, which makes comparisons between our cohort and community-based cohorts [28] difficult.

## 6. Conclusions

This study aimed to shed more light on the clinical dilemma between the high risk of stroke on the one hand and potentially life-threatening bleeding events on the other. For the first time, we confirm that OAC-treated HFpEF patients are at risk of GI bleeding and demonstrate that high RAP is predictive for GI bleeding events in this specific patient population. Although confirmation from larger trials is currently lacking, our data suggest that beyond the well-established HAS-BLED score, RAP or its surrogates can be taken into account to define patients’ individual bleeding risk when initiating OAC treatment in this high-risk HF population.

## Figures and Tables

**Table 1:** Baseline characteristics of registered patients.

Variable *n* (%)	All Patients (*n* = 328)	No. OAC (*n* = 116)	OAC (*n* = 212)	*p*-Value
**Clinical parameters**				
Age, years (IQR)	71 (67–77)	71 (65–76)	73 (68–77)	**0.026**
Female gender, *n* (%)	233 (71)	92 (75)	141 (69)	0.179
Body mass index, kg/m^2^ (IQR)	31 (26–34)	29 (25–35)	30 (27–34)	0.470
6-min walk distance, m (IQR)	329 (229–413)	340 (240–434)	326 (227–390)	0.110
Systolic blood pressure, mmHg (IQR)	141 (125–155)	142 (130–160)	140 (124–150)	**0.005**
Diastolic blood pressure, mmHg (IQR)	80 (70–89)	80 (70–90)	80 (70–86)	0.289
NYHA functional class ≥ III, *n* (%)	183 (55.8)	59 (49.6)	124 (62.3)	**0.030**
NT-proBNP, pg/mL (IQR)	1083 (422–2002)	548 (303–1069)	1389 (700–2286)	**<0.001**
Antiplatelet therapy, *n* (%)	97 (29.6)	68 (58.1)	29 (13.7)	**<0.001**
Antithrombotic therapy with NOAC, *n* (%)	74 (34.9)	-	74 (34.9)	
Antithrombotic therapy with VKA, *n* (%)	138 (65.1)	-	138 (65.1)	
**Co-morbidities**				
Atrial fibrillation, *n* (%)	192 (58.5)	18 (14.8)	174 (84.5)	**<0.001**
CHA_2_DS_2_-VASc score, median (IQR)	5 (4–6)	4 (3–5)	5 (4–6)	**<0.001**
HAS-BLED score, median (IQR)	3 (2–4)	3 (2–3)	3 (2–4)	**0.635**
Bleeding events, *n* (%)	54 (16.5)	5 (4.3)	49 (23.1)	**0.003**
Thromboembolic events, *n* (%)	6 (2.0)	1 (1.0)	5 (2.5)	0.355
Arterial hypertension, *n* (%)	311 (94.8)	114 (93.4)	197 (96.1)	0.282
Chronic kidney disease *, *n* (%)	138 (44.5)	35 (32.1)	103 (51.2)	**0.001**
Diabetes mellitus, *n* (%)	117 (35.7)	45 (36.9)	72 (35.5)	0.797
Anemia, *n* (%)	193 (61.9)	73 (65.8)	120 (59.7)	0.291
**Invasive hemodynamic parameters**				
Mean pulmonary arterial pressure, mmHg (IQR)	33 (26–39)	32 (25–38)	33 (28–39)	**0.049**
Right atrial pressure, mmHg (IQR)	12 (8–16)	11 (8–15)	12 (9–16)	0.112
Pulmonary artery wedge pressure, mmHg (IQR)	19 (16–23)	18 (15–22)	20 (17–24)	**0.006**
Left ventricular end diastolic pressure, mmHg (IQR)	19 (16–23)	20 (15–25)	19 (16–23)	0.415
Stroke volume index, mL/m^2^ (IQR)	70.0 (59.0–86.9)	71.5 (62.7–85.7)	68.00 (56.4–87.2)	0.160
Cardiac index, L/min/m^2^ (IQR)	2.7 (2.3–3.1)	2.8 (2.5–3.4)	2.7 (2.2–3.0)	**0.011**
Pulmonary vascular resistance, dyn·s·cm^−5^ (IQR)	200.5 (141.2–284.6)	204.9 (141.8–263.3)	199.0 (141.2–290.9)	0.812

OAC, oral anticoagulation; NYHA, New York Heart Association; NT-proBNP, N-terminal prohormone of brain natriuretic peptide; NOAC, non-vitamin K oral anticoagulant; VKA, vitamin K antagonist; CHA_2_DS_2_-VASc (Congestive heart failure, Hypertension, Age ≥75 years, Diabetes mellitus, prior Stroke, Vascular disease, Age 65–74 years, Sex category) Score calculates stroke risk for patients with atrial fibrillation; HAS-BLED (Hypertension, Abnormal Renal/Liver Function, Stroke, Bleeding History or Predisposition, Labile INR, Elderly, Drugs/Alcohol Concomitantly) Score estimates risk for major bleeding; RV, right ventricular. Values are given as median and interquartile range (IQR), or total numbers (*n*) and percent (%). Bold indicates *p* < 0.05. * Estimated glomerular filtration rate <60 mL/min/1.73m^2^.

**Table 2:** Clinically relevant bleeding events in orally anticoagulated patients versus the remainder of the study cohort.

Variable *n* (%)	All Patients (*n* = 328)	No OAC (*n* = 116)	OAC (*n* = 212)	*p*-Value	NOAC (*n* = 74)	VKA (*n* = 138)	*p*-Value
Bleeding events, *n* (%)	54 (16.5)	5 (4.3)	49 (23.1)	**<0.001**	16 (21.6)	33 (23.9)	**<0.001**
Cerebral bleeding, *n* (%)	4 (1.2)	0 (0.0)	4 (1.9)	0.137	0 (0.0)	4 (2.9)	0.062
Gastrointestinal bleeding, *n* (%)	21 (6.4)	3 (2.6)	18 (8.5)	**0.037**	6 (8.1)	12 (8.7)	0.111
Urogenital bleeding, *n* (%)	5 (1.5)	1 (0.9)	4 (1.9)	0.469	1 (1.4)	3 (2.2)	0.690
Hematoma bleeding, n (%)	8 (2.4)	0 (0.0)	8 (3.8)	**0.034**	5 (6.8)	3 (2.2)	**0.013**
Nasal bleeding, *n* (%)	14 (4.3)	1 (0.9)	13 (6.1)	**0.024**	3 (4.1)	10 (7.2)	**0.043**
Other bleeding, *n* (%)	2 (0.6)	0 (0.0)	2 (0.9)	0.294	1 (1.4)	1 (0.7)	0.493

OAC, oral anticoagulation; NOAC, non-vitamin K oral anticoagulant; VKA, vitamin K antagonist Clinically relevant bleeding events were defined in accordance to the definition of major bleeding recommended by the International Society on Thrombosis and Haemostasis [22]. Major bleeding was defined as fatal bleeding and/or bleeding into a critical organ (intracranial, intraocular, retroperitoneal, intraarticular, pericardial, or intramuscular) and/or clinically relevant bleeding with a drop in hemoglobin ≥ 2 g/dL or requiring to transfusion. Values are given as total numbers (*n*) and percent (%). Bold indicates *p* < 0.05.

**Table 3:** Cox proportional hazard model of patients treated with oral anticoagulation (*n* = 212) with regard to overall bleeding events (*n* = 49).

Variable	Hazard Ratio	95% Confidence Interval	*p-*Value	Adjusted Hazard Ratio	95% Confidence Interval	*p-*Value
**Clinical parameters**						
Systolic blood pressure, mmHg	1.01	(1.00–1.03)	0.103			
Diastolic blood pressure, mmHg	1.00	(0.97–1.02)	0.671			
Antithrombotic therapy with VKA	1.31	(0.70–2.47)	0.399			
Antiplatelet therapy	1.51	(1.00–1.76)	0.05			
**Co-morbidities**						
CHA_2_DS_2_-VASc score	1.07	(0.88–1.31)	0.477			
HAS-BLED score	2.14	(1.66–2.74)	**<0.00**1	2.61	(1.92–3.55)	**<0.001**
**Invasive hemodynamic parameters**						
Mean pulmonary arterial pressure, mmHg	1.01	(0.99–1.04)	0.345			
Mean right atrial pressure, mmHg	1.05	(1.01–1.11)	**0.031**	1.02	(0.95–1.09)	0.598
Pulmonary artery wedge pressure, mmHg	1.06	(1.00–1.12)	**0.041**	1.05	(0.98–1.12)	0.193
Left ventricular end diastolic pressure, mmHg	1.06	(0.99–1.12)	0.078			

VKA, vitamin K antagonist; CHA_2_DS_2_-VASc (Congestive heart failure, Hypertension, Age ≥75 years, Diabetes mellitus, prior Stroke, Vascular disease, Age 65–74 years, Sex category) Score calculates stroke risk for patients with atrial fibrillation; HAS-BLED (Hypertension, Abnormal Renal/Liver Function, Stroke, Bleeding History or Predisposition, Labile INR, Elderly, Drugs/Alcohol Concomitantly) score estimates risk for major bleeding; Bold indicates *p* < 0.05.

**Table 4:** Cox proportional hazard model of patients treated with oral anticoagulation (*n* = 212) with regard to gastrointestinal bleeding events (*n* = 18).

Variable	Hazard Ratio	95% Confidence Interval	*p*-Value	Adjusted Hazard Ratio	95% Confidence Interval	*p*-Value
**Clinical parameters**						
Systolic blood pressure, mmHg	1.01	(0.98–1.03)	0.66			
Diastolic blood pressure, mmHg	1.04	(1.00–1.08)	**0.044**	1.04	(1.03–1.10)	0.292
Antithrombotic therapy with NOAC	0.43	(0.14–1.36)	0.152			
Antiplatelet therapy	0.97	(0.92–1.02)	0.204			
**Co-morbidities**						
CHA_2_DS_2_-VASc score	0.83	(0.60–1.14)	0.252			
HAS-BLED score	1.96	(1.29–2.99)	**0.002**	1.74	(1.15–2.64)	**0.009**
**Invasive hemodynamic parameters**						
Mean pulmonary arterial pressure mmHg	1.05	(1.01–1.09)	**0.015**	0.95	(0.87–1.03)	0.195
Right atrial pressure mmHg	1.13	(1.06–1.20)	**<0.001**	1.13	(1.03–1.25)	**0.01**3
Pulmonary artery wedge pressure mmHg	1.19	(1.09–1.30)	**<0.001**	1.11	(0.96–1.28)	0.161
Left ventricular end diastolic pressure mmHg	1.09	(1.01–1.19)	**0.035**	1.05	(0.93–1.19)	0.439

NOAC, non-vitamin K oral anticoagulant; CHA_2_DS_2_-VASc (Congestive heart failure, Hypertension, Age ≥75 years, Diabetes mellitus, prior Stroke, Vascular disease, Age 65–74 years, Sex category) Score calculates stroke risk for patients with atrial fibrillation; HAS-BLED (Hypertension, Abnormal Renal/Liver Function, Stroke, Bleeding History or Predisposition, Labile INR, Elderly, Drugs/Alcohol Concomitantly) Score estimates risk for major bleeding; Bold indicates *p* < 0.05.

## Supplementary Materials

The following are available online at https://www.mdpi.com/2077-0383/8/8/1240/s1: Table S1: Additional baseline characteristics of registered patients. Table S2: Cox proportional hazard model of registered patients with regard to all bleeding events. Table S3: Baseline characteristics of orally anticoagulated patients with regard to gastrointestinal bleeding.

## Abbreviations

AF	atrial fibrillation
ALAT	alanine aminotransferase
ASAT	aspartate aminotransferase
AUC	area under the curve
CHA_2_DS_2_-VASc score	Congestive heart failure, Hypertension, Age ≥75 years, Diabetes mellitus, prior Stroke or TIA or thromboembolism, Vascular disease (e.g., peripheral artery disease, myocardial infarction, aortic plaque), Age 65-74 years and Sex category (i.e., female sex)
CI	confidence interval
CMR	cardiac magnetic resonance
E/e’	ratio of early transmitral blood velocity to early diastolic mitral annular velocity
Gamma-GT	gamma-glutamyltransferase
GI	gastrointestinal
HFpEF	heart failure with preserved ejection fraction
HAS-BLED	Hypertension, Abnormal Renal/Liver Function, Stroke, Bleeding History or Predisposition, Labile INR, Elderly, Drugs/Alcohol Concomitantly
HF	heart failure
HR	hazard ratio
IDI	integrated discrimination improvement
IQR	interquartile range
LV	left ventricular
LVEDP	LV enddiastolic pressure
NOAC	non-vitamin K oral anticoagulant
NRI	net reclassification improvement
NYHA	New York Heart Association
NT-proBNP	serum N-terminal pro–B-type natriuretic peptide
OAC	oral anticoagulation
PAWP	pulmonary arterial wedge pressure
dPAP	diastolic pulmonary artery pressure
mPAP	mean pulmonary artery pressure
sPAP	systolic pulmonary artery pressure
mRAP	mean right atrial pressure
RHC	right heart catheterization
RV	right ventricular
TTE	transthoracic echocardiography
VKA	vitamin K antagonist
