# Supplementary material for: Patients with Heart Failure and Preserved Ejection Fraction Are at Risk of Gastrointestinal Bleeding
Source: J Clin Med. 2019 Aug 17;8(8):1240. doi: 10.3390/jcm8081240 (PMC6724012; doi:10.3390/jcm8081240)
Supplement: Supplementary file 1 [file jcm-08-01240-s001.pdf]

Journal of
Clinical Medicine

MDPI

Supplementary Tables

Table S1. Additional baseline characteristics of registered patients.

Variable n (%)	All patients (n = 328)	No OAC (n = 116)	OAC (n = 212)	*p* value
**Laboratory parameters**				
Platelet count, G/L (IQR)	224 (187–266)	226 (196–263)	222 (181–267)	0.471
Albumin, g/L (IQR)	41.2 (38.7–43.2)	41.3 (37.95–42.90)	41.20 (38.90–43.30)	0.319
**Cholinesterase, kU/L (IQR)**	6.40 (5.13–8.02)	7.79 (5.83–9.15)	6.21 (5.00–7.44)	**0.030**
ALAT, U/L (IQR)	21 (16–28)	21 (15–25)	22 (16–30)	0.111
**ASAT, U/L (IQR)**	25 (20–31)	23 (20–28)	25 (21–33)	**0.001**
**Gamma–GT, U/L (IQR)**	34 (22–66)	26 (17–43)	41 (27–77)	**<0.001**
**Serum Creatinine, mg/dL (IQR)**	1.06 (0.87–1.31)	0.98 (0.82–1.15)	1.13 (0.91–1.34)	**0.003**
**Echocardiographic parameters**				
E/e′, ratio (IQR)	15 (11–20)	14.5 (10.4–20.4)	15 (11–20)	0.802
**Left atrial diameter, mm (IQR)**	61 (57–66)	58 (54–62)	64 (60–68)	**<0.001**
**Right atrial diameter, mm (IQR)**	62 (56–68)	57 (52–62)	64 (58–70)	**<0.001**
**RV–performance moderately to severely reduced n (%)**	40 (13.6)	5 (4.1)	35 (17.0)	**0.001**
**Systolic pulmonary arterial pressure, mmHg (IQR)**	56 (44–70)	48 (38–62)	59 (48–74)	**<0.001**
**Significant tricuspid regurgitation‡, n (%)**	175 (54.5)	43 (35.2)	132 (64.1)	**<0.001**
**Cardiac magnetic resonance imaging parameters**				
**Left atrial area index, mm/m² (IQR)**	30 (25–36)	27 (23–33)	32 (27–37)	**<0.001**
**Right atrial area index, mm/m² (IQR)**	27 (23–34)	24 (21–28)	30 (25–36)	**<0.001**
**Left ventricular ejection fraction, % (IQR)**	27 (23–34)	67 (60–74)	61 (53–71)	**0.009**
Left ventricular end-diastolic volume index, mL/m² (IQR)	63 (55–72)	118 (102–136)	120 (101–147)	0.617
Left ventricular mass index, g/m² (IQR)	103 (102–146)	98 (83–135)	106 (89–133)	0.257
**Right ventricular ejection fraction, % (IQR)**	52 (45–61)	55 (47–63)	50 (44–60)	**0.022**
Right ventricular end-diastolic volume index, mL/m² (IQR)	141 (115–182)	130 (112–166)	146 (119–187)	0.056

OAC, oral anticoagulation; ALAT, alanine aminotransferase; ASAT, aspartate aminotransferase; Gamma-GT, gamma-glutamyltransferase; E/e′, ratio of early transmitral blood velocity to early diastolic mitral annular velocity; RV, right ventricular. Values are given as median and interquartile range (IQR), or total numbers (n) and percent (%). Bold indicates $p < 0.05$. * Estimated glomerular filtration rate <60mL/min/1.73m².

Table S2. Cox proportional hazard model of registered patients with regard to all bleeding events.

Variable	Hazard ratio	95% Confidence nterval	*p* value	Adjusted hazard ratio	95% Confidence interval	*p* value
**Clinical parameters**						
Systolic blood pressure, mmHg	1.01	(1.00–1.03)	0.101			
Diastolic blood pressure, mmHg	0.99	(0.97–1.02)	0.54			
**Antithrombotic therapy with VKA**	1.44	(1.03–1.68)	**0.038**	1.80	(1.50–1.92)	**0.001**
**Antithrombotic therapy with NOAC**	1.54	(1.17–1.75)	**0.010**	7.13	(2.60–19.53)	**<0.001**
**Antiplatelet therapy**	1.52	(1.07–1.75)	**0.029**	1.36	(0.69–1.35)	0.245
**Co-morbidities**						
CHA_2DS_2-VASc score	0.95	(0.79–1.15)	0.615			
**HAS-BLED score**	2.12	(1.66–2.70	**<0.001**	2.23	(1.72–2.89)	**<0.001**
**Invasive hemodynamic parameters**						
Mean pulmonary arterial pressure, mmHg	1.00	(0.98–1.03)	0.815			
Right atrial pressure, mmHg	1.04	(0.99–1.09)	0.087			
Pulmonary artery wedge pressure, mmHg	1.05	(1.00–1.11)	0.061			
Left ventricular end diastolic pressure, mmHg	1.05	(0.99–1.12)	0.085			

NOAC, non-vitamin K oral anticoagulant; VKA, vitamin K antagonist; CHA_2DS_2-VASc (Congestive heart failure, Hypertension, Age ≥75 years, Diabetes mellitus, prior Stroke, Vascular disease, Age 65-74 years, Sex category) Score calculates stroke risk for patients with atrial fibrillation; HAS-BLED (Hypertension, Abnormal Renal/Liver Function, Stroke, Bleeding History or Predisposition, Labile INR, Elderly, Drugs/Alcohol Concomitantly) Score estimates risk for major bleeding; ALAT, alanine aminotransferase; ASAT, aspartate aminotransferase; Gamma-GT, gamma-glutamyltransferase; E/e', ratio of early transmitral blood velocity to early diastolic mitral annular velocity; RV, right ventricular Bold indicates $p<0.05$.

Table 3. Baseline characteristics of orally anticoagulated patients with regard to gastrointestinal bleeding.

Variable N (%)	All patients on OAC (n=212)	No GI bleeding (n=194)	GI bleeding (n=18)	*p* value
**Clinical parameters**				
Age, years (IQR)	72 (68–77)	72 (68–11)	69 (64–75)	0.326
Female gender, n (%)	144 (67.9)	134 (69.1)	10 (55.6)	0.240
**Body mass index, kg/m² (IQR)**	30.0 (26.1–33.9)	29.6 (26.0–32.4)	34.1 (33.6–34.9)	**0.018**
**6-minute walk distance, m (IQR)**	310 (227–390)	317 (247–400)	243 (100–329)	**0.032**
Systolic blood pressure, mmHg (IQR)	138 (122–150)	138 (123–150)	138 (114–160)	0.794
Diastolic blood pressure, mmHg (IQR)	79 (70–85)	79 (70–86)	74 (61–85)	0.930
NYHA functional class ≥ III, n (%)	129 (62.9)	117 (61.9)	12 (75.0)	0.425
NT-proBNP, pg/mL (IQR)	1389 (700–2286)	1066 (420–1979)	1348 (456–2695)	0.789
**Antiplatelet therapy, n (%)**	29 (14.0)	167 (87.4)	11 (68.8)	**0.039**
**Co-morbidities**				
**Atrial fibrillation, n (%)**	179 (84.4)	167 (86.1)	12 (66.7)	**0.030**
CHA_2DS_2-VASc score, median (IQR)	5.00 (4.00–6.00)	5.00 (4.00–6.00)	4.50 (4.00–6.25)	0.289
**HAS-BLED score, median (IQR)**	3.00 (2.00–4.00)	3.00 (2.00–4.00)	4.00 (3.00–5.00)	**<0.001**
Arterial hypertension, n (%)	202 (95.7)	184 (95.3)	18 (100)	0.349
**Chronic kidney disease*, n (%)**	106 (51.2)	92 (48.7)	14 (77.8)	**0.018**
Diabetes mellitus, n (%)	74 (35.4)	69 (36.1)	5 (27.8)	0.479
Anemia, n (%)	124 (59.9)	111 (58.1)	13 (81.3)	0.070
**Laboratory parameters**				
Platelet count, G/L (IQR)	233.21 (81.00–266.00)	130.02 (181.00–267.00)	267.61 (182.00–246.00)	0.773
Albumin, g/L (IQR)	41.16 (38.70–43.20)	41.04 (38.70–43.20)	42.38 (38.30–43.40)	0.754
Cholinesterase, kU/L (IQR)	6.24 (5.12–7.43)	6.48 (5.16–7.53)	5.57 (4.44–6.35)	0.624
ALAT, U/L (IQR)	25.69 (16.00–31.00)	26.06 (17.00–31.00)	21.72 (14.00–27.00)	0.085
ASAT, U/L (IQR)	27.85 (21.00–33.00)	27.84 (21.00–33.00)	28.00 (23.00–31.00)	0.838
Gamma-GT, U/L (IQR)	76.16 (26.00–78.50)	75.50 (26.00–80.00)	83.22 (28.00–75.00)	0.411
Serum Creatinine, mg/dL (IQR)	1.23 (0.91–1.35)	1.19 (0.90–1.33)	1.73 (1.02–1.70)	0.203
**Echocardiographic parameters**				
E/e', ratio (IQR)	15.5 (11.0–18.3)	15.5 (11.0–20.0)	16.0 (15.0–18.3)	0.705
Left atrial diameter, mm (IQR)	64 (60–68)	64 (60–68)	62 (56–69)	0.491
Right atrial diameter, mm (IQR)	64 (58–70)	64 (58–70)	65 (58–71)	0.992
RV-performance moderately to severely reduced n (%)	35 (16.5)	30 (15.5)	5 (27.8)	0.178
Systolic pulmonary arterial pressure, mmHg (IQR)	61 (48–74)	61 (48–72)	67 (54–75)	0.070
**Significant tricuspid regurgitation**, n (%)**	15 (7.7)	11 (6.3)	4 (22.2)	**0.016**

**Cardiac magnetic resonance imaging parameters**				
Left atrial area index, mm/m² (IQR)	34 (27–37)	34 (27–37)	32 (27–36)	0.732
Right atrial area index, mm/m² (IQR)	32 (25–36)	32 (25–36)	29 (24–35)	0.930
Left ventricular ejection fraction, %	62 (53–71)	62 (53–71)	65 (57–76)	0.664
Left ventricular end-diastolic volume index, mL/m² (IQR)	128 (100–147)	126 (99–142)	157 (114–197)	0.698
Left ventricular mass index, g/m² (IQR)	111 (89–133)	11 (89–133)	118 (92–151)	0.374
Right ventricular ejection fraction, %	51 (44–60)	51 (44–60)	54 (43–63)	0.664
Right ventricular end-diastolic volume index, mL/m² (IQR)	165 (117–187)	164 (117–186)	187 (124–195)	0.732
**Invasive hemodynamic parameters**				
**Mean pulmonary arterial pressure, mmHg (IQR)**	35 (28–39)	34 (27–38)	41 (34–48)	**0.040**
**Right atrial pressure, mmHg (IQR)**	12 (8–16)	12 (7–15)	19 (16–21)	**<0.001**
**Pulmonary artery wedge pressure, mmHg (IQR)**	20 (17–24)	19 (16–23)	26 (22–31)	**0.005**
Left ventricular end diastolic pressure, mmHg (IQR)	19 (16–23)	19 (15–22)	23 (19–28)	0.119
Stroke volume index, mL/m² (IQR)	38.99 (30.83–46.12)	38.51 (30.80–45.48)	43.37 (34.27– 47.62)	1.000
Cardiac index, L/min/m² (IQR)	2.70 (2.20–3.00)	2.68 (2.18–2.97)	2.88 (2.54–3.43)	0.587
Pulmonary vascular resistance, dyn·s·cm⁻⁵ (IQR)	235.14 (140.63–290.91)	231.22 (141.23–286.91)	271.67 (126.32–425.53)	0.979

OAC, oral anticoagulation; NYHA, New York Heart Association; NT-proBNP, N-terminal prohormone of brain natriuretic peptide; NOAC, non-vitamin K oral anticoagulant; VKA, vitamin K antagonist; CHA_2DS_2-VASc (Congestive heart failure, Hypertension, Age ≥75 years, Diabetes mellitus, prior Stroke, Vascular disease, Age 65-74 years, Sex category) Score calculates stroke risk for patients with atrial fibrillation; HAS-BLED (Hypertension, Abnormal Renal/Liver Function, Stroke, Bleeding History or Predisposition, Labile INR, Elderly, Drugs/Alcohol Concomitantly) Score estimates risk for major bleeding; ALAT, alanine aminotransferase; ASAT, aspartate aminotransferase; Gamma-GT, gamma-glutamyltransferase; E/e′, ratio of early transmitral blood velocity to early diastolic mitral annular velocity; RV, right ventricular. Values are given as median and interquartile range (IQR), or total numbers (n) and percent (%). Bold indicates $p<0.05$. * Estimated glomerular filtration rate <60mL/min/1.73m².
